# Longitudinal study reveals plasma glycans associations with prediabetes/type 2 diabetes in KORA study

**DOI:** 10.1186/s12933-025-02853-y

**Published:** 2025-08-06

**Authors:** Jiefei Niu, Elke Rodriguez, Tamara Štambuk, Irena Trbojević-Akmačić, Nikol Mraz, Jochen Seissler, Thomas Skurk, Sabrina Schlesinger, Annette Peters, Gordan Lauc, Christian Gieger, Harald Grallert

**Affiliations:** 1 Research Unit of Molecular Epidemiology, Helmholtz Zentrum München, 85764 Neuherberg, Germany; 2 Institute of Epidemiology, Helmholtz Zentrum München, 85764 Neuherberg, Germany; 3 Faculty of Medicine, Ludwig-Maximilians-University München, 81377 Munich, Germany; 4 German Center for Diabetes Research (DZD), 85764 Neuherberg, Germany; 5 Glycoscience Research Laboratory, Genos Ltd, Borongajska 83H, 10000 Zagreb, Croatia; 6 Chair of Epidemiology, Faculty of Medicine, Ludwig-Maximilians-University München, 81377 Munich, Germany; 7 School of Medicine, Technical University of Munich, 81675 Munich, Germany; 8 ZIEL Institute for Food and Health, Core Facility Human Studies, Technical University of Munich, 85354 Freising, Germany; 9 Medizinische Klinik und Poliklinik IV, Klinikum der Universität München, Ludwig-Maximilians-University München, 80336 Munich, Germany; 10 Institute for Biometrics and Epidemiology, German Diabetes Center, Leibniz Center for Diabetes Research at Heinrich Heine University Düsseldorf, 40225 Düsseldorf, Germany; 11 German Center for Diabetes Research (DZD), Partner Düsseldorf, Muenchen-Neuherberg, 40225 Düsseldorf, Germany; 12 Faculty of Pharmacy and Biochemistry, University of Zagreb, 10000 Zagreb, Croatia

**Keywords:** Plasma N-glycome, Glycomics, Diabetes, Prediabetes, Machine learning, Glycan-QTL

## Abstract

**Background:**

Altered plasma N-glycosylation is increasingly recognized as a contributor to metabolic dysregulation. This study aimed to investigate the role of plasma N-glycans in glucose metabolism and the progression from normoglycemia to prediabetes and type 2 diabetes (T2D).

**Methods:**

We analyzed longitudinal data from 473 participants in the Cooperative Health Research in the Region of Augsburg (KORA) cohort over 7 years. N-glycan profiles were measured using hydrophilic interaction ultrahigh-performance liquid chromatography with fluorescence detection (HILIC-UHPLC-FLR). Glycan associations with incident prediabetes/T2D and related traits, such as body mass index (BMI), fasting glucose, homeostasis model assessment of insulin resistance (HOMA-IR) were evaluated using longitudinal models based on N-glycan measurements obtained at F4 and FF4. Classification performance at FF4 was assessed using machine learning models interpreted with SHapley Additive exPlanation (SHAP) values. Mendelian randomization (MR) and glycan quantitative trait loci (glycan-QTL) analyses were conducted to explore causality and genetic determinants.

**Results:**

During follow-up, 231 individuals progressed to prediabetes/T2D, while 242 remained normoglycemic. Nineteen glycans were associated with diabetes progression in the basic model; 12 remained significant after full adjustment. Glycans such as GP18 and GP32 were also linked to metabolic traits. A glycan-clinical model achieved high classification accuracy (AUC = 0.895). MR supported causal roles for GP18, GP19, and S1. Glycan-QTL analysis revealed SNPs and genes (FUT8, ST3GAL4) are associated with key glycans.

**Conclusions:**

Plasma N-glycans are diagnostic of early glycemic deterioration and supported by genetic and causal evidence, highlighting their potential as biomarkers for diabetes risk stratification.

**Graphic abstract:**

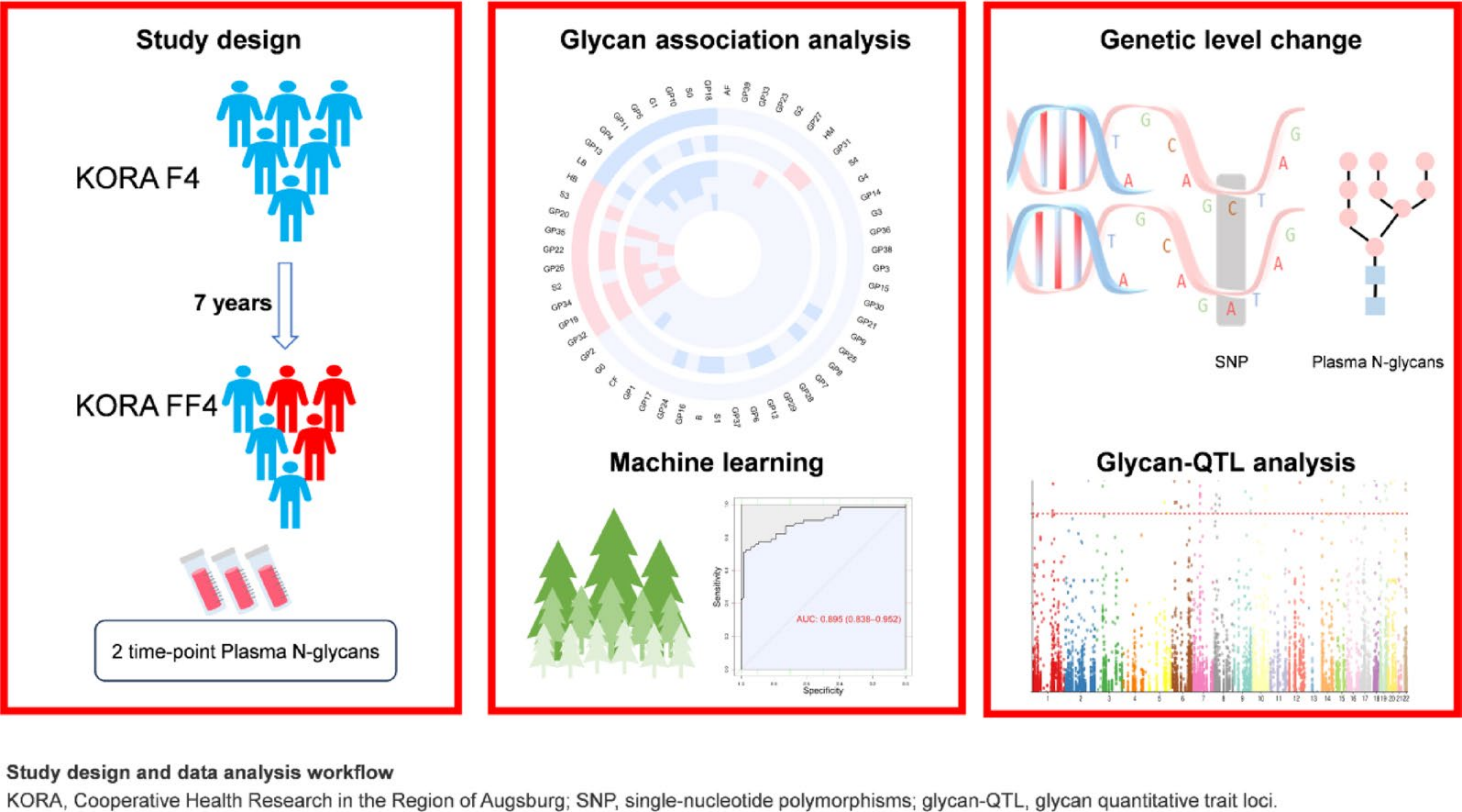

**Supplementary Information:**

The online version contains supplementary material available at 10.1186/s12933-025-02853-y.

## Background

Diabetes has emerged as a global health burden owing to its high incidence, associated disability, and mortality. It is estimated to be the eighth leading cause of death and disability worldwide [1, 2]. Approximately 537 million adults are affected by diabetes globally, with the majority having type 2 diabetes (T2D). This figure is projected to increase to 783 million by 2045 [3]. Furthermore, approximately 352 million individuals are diagnosed with impaired fasting glucose (IFG) or impaired glucose tolerance (IGT) [4] conditions that have the potential to progress to T2D at an annual rate of 5–10% within this population [5]. For years, venous blood glucose and laboratory-based hemoglobin A1c (HbA1c) have served as standard biomarkers and diagnostic tools for diabetes screening. However, research continues to explore new potential biomarkers. Clinically validated biomarkers, such as glycated albumin (GA) and fructosamine (FA), have demonstrated high specificity and sensitivity for diabetes diagnosis. These biomarkers serve as valuable alternatives when HbA1c measurements are not feasible [6]. Additionally, novel markers identified through metabolomics, including fetuin-A, branched-chain amino acids (BCAAs), adipokines, linoleoylglycerophosphocholine (L-GPC), and lysophosphatidylcholine (LysoPC), have shown strong correlations with HbA1c and blood glucose levels, suggesting their potential as future diagnostic tools [6, 7]. Although there are numerous T2D biomarkers, many are based on pathophysiological changes. There remains a significant need for biomarkers that can identify at-risk individuals at earlier stages, such as individuals with prediabetes or even those with normal glucose tolerance (NGT).

N-glycosylation is the most prevalent type of protein glycosylation that is enzymatically mediated by glycosyltransferases. It involves the attachment of glycans, which are complex carbohydrate molecules, to the asparagine residues of proteins [8]. Glycosylation alters and regulates the biological activity of many proteins, significantly contributing to their functional diversity [9]. As glycan biosynthesis is highly responsive to the cellular state and environment, altered glycosylation may reflect various pathophysiological changes [10]. Moreover, the composition of plasma protein N-glycans within healthy individuals is remarkably stable [11]. The role of plasma protein N-glycans in diabetes development has been highlighted in several previous studies, however, most of these were cross-sectional in design or involved glycomic profiling at a single time point, limiting their ability to capture glycan dynamics during disease progression. For example, in a case‒control study of 562 T2D patients and 599 healthy controls, α(1,6)-linked arm monogalactosylated, core-fucosylated diantennary plasma N-glycans (FA2[6]G1) were significantly reduced in T2D patients compared with controls, regardless of complications [12]. Similarly, increased α1,6-fucosylation of N-glycans has been observed in *db/db* mice, a model of T2D with obesity [13]. Moreover, the TwinsUK study suggested that plasma N-glycan changes might serve as early indicators of diabetes, identifying individuals at risk before clinical symptoms arise [14] and have also shown that selected plasma N-glycans improve T2D prediction beyond established risk markers [15].

To address these limitations, our study is the first to utilize two-time-point longitudinal glycomic data from the KORA F4/FF4 cohort. F4 consists of healthy individuals, and FF4 includes individuals diagnosed with prediabetes/T2D and healthy controls. This study aimed to identify plasma N-glycans associated with the incidence of prediabetes or T2D in 473 individuals (231 individuals with incident prediabetes/T2D, 242 controls), providing a unique opportunity to explore how plasma N-glycan profiles associated with prediabetes/T2D change and evolve. Additionally, we explored the relationships between the plasma N-glycome and disease diagnosis, causal relationships, and genetic-level alterations.

## Methods

### Study population

The KORA study is a population-based German cohort. KORA S4 (1999–2001) enrolled 4261 adults (25–74 years) with German citizenship in Augsburg. The follow-ups included KORA F4 (2006–2008, *n* = 3080) and KORA FF4 (2013–2014, *n* = 2,279). The study was approved by the Ethics Committee of the Bavarian Chamber of Physicians, all procedures followed the ethical standards of the Declaration of Helsinki. All participants provided written and informed consent. More details of the study design have been described previously in detail [16].

In this study, we utilized KORA F4 and FF4 to design a matched case‒control study. We analyzed the plasma protein N-glycome in 1000 plasma samples from KORA F4/FF4 via hydrophilic interaction ultrahigh-performance liquid chromatography with fluorescence detection (HILIC-UHPLC-FLR). From participants with paired plasma samples at both F4 and FF4, we identified 250 cases who progressed from NGT to prediabetes (n = 150) or T2D (n = 100). Controls with persistent NGT were 1:1 age (± 2 years)-, sex-matched, and randomly selected from eligible individuals. Subjects with fever, infections, or acute gastrointestinal diseases were excluded from the test. After excluding 26 individuals with missing values, such as HbA1c, homeostasis model assessment of insulin resistance (HOMA-IR), urine albumin-to-creatinine ratio and insufficient fasting duration (< 8 h, *n* = 1), 473 participants remained for analysis (NGT = 242, prediabetes/T2D = 231) (Supplementary Fig. 1). The World Health Organization diagnostic criteria were applied to the classification of KORA participants. Prediabetes comprises isolated IFG, isolated IGT, and combined IFG and IGT, details are provided in Supplementary Methods.

### Clinical measurements and assessments of risk factors

All participants underwent standard physical and medical examinations at KORA F4 and FF4. A detailed description of the measurement methods can be found in the Supplementary Methods.

### Plasma N-glycome measurements

The plasma samples (10 μL) were denatured by adding 20 μL of 2% (w/v) sodium dodecyl sulfate (SDS, Invitrogen, USA) and incubating at 65 °C for 10 min. After adding 10 μL of 4% (v/v) Igepal CA-630 (Sigma‒Aldrich, USA), the mixture was shaken for 15 min, followed by 18 h of incubation at 37 °C with 1.2 U of PNGase F (Promega, USA) to release the N-glycans. The released N-glycans were labeled with the fluorescent dye 2-aminobenzamide (2-AB). Solid-phase extraction (SPE) was then performed via HILIC to purify the labeled glycans. The labeled N-glycans were separated via HILIC-UHPLC-FLR, and the chromatograms were all separated in the same manner into 39 plasma glycan peaks (GPs). In addition to the 39 glycan peaks, 16 derived traits representing glycosylation features were calculated. More details on the glycoanalytical procedure are reported in the Supplementary Methods. A complete description of the glycan structures, derived trait calculation formulas, and reference data sources is provided in Supplementary Tables 1 and 2.

### Statistical analysis

#### Glycan data normalization and batch correction

Raw glycan peak data, expressed as areas under the curve, were normalized to reduce experimental noise by dividing each peak area by the total integrated area of all peaks, multiplying by 100 to represent percentages, and enabling comparisons across samples regardless of intensity differences. To further address experimental variation during sample preparation and analysis, batch correction was applied to the normalized data. After the data were log-transformed to correct for right-skewed distributions, the ComBat method from the R package ‘sva’ was used, incorporating the sample plate order as a batch covariate. Batch correction was performed for each glycan peak individually. The values were subsequently transformed back to the original scale.

#### Characteristics of KORA F4/FF4

The characteristics of the study population are presented as mean ± standard deviations (SDs) or medians (25th and 75th percentiles) for continuous variables with normal or nonnormal distributions, respectively. Categorical variables are presented as frequencies (percentages).

#### Longitudinal analysis of the 2 time points

General estimating equation (GEE) [17] models were used to identify glycan structures that were significantly different in individuals before the diagnosis of prediabetes or T2D compared with controls. For each glycan, the values were transformed to a standard normal distribution before fitting the models via the R package GenABEL [18]. Disease status was used as an independent variable, adjusting for additional covariates, such as age, sex, and BMI, and was included in the basic model. In the full model, we also included smoking status, alcohol consumption, physical activity, high-density lipoprotein (HDL), and systolic blood pressure (SBP). This process was repeated for the 39 glycan peaks and 16 derived glycan traits. In the sensitivity analysis, we compared differences in glycan levels between the subgroups NGT and prediabetes, NGT and T2D, prediabetes and T2D to evaluate glycan alterations across different stages of glucose metabolism dysregulation. The associations of BMI and T2D-related traits (fasting plasma glucose (FPG), HOMA-IR, and HbA1c) with each plasma glycan were tested via a linear mixed model (LMM), adjusting for both the basic and full models. In the LMM, the individual ID was considered a random effect. For multiple testing via the Bonferroni method, an adjusted *p* < 0.05 was considered significant.

#### Glycan-based diagnostic modeling and explainability at FF4

The discriminatory potential of individual glycan peaks was further evaluated via the area under the curve (AUC). The set of 19 glycan peaks was significantly associated with disease status in the previous step. The random forest (RF) model [19] was used to construct diagnostic classification models for the KORA FF4 dataset. Tenfold cross-validation was employed to tune the model's parameters. Three models including different sets of features were developed for comparison, including a 19-glycan-only model, the Framingham Offspring Risk Score (FORS) model [20], and a combined 19-glycan + FORS model. The receiver operating characteristic (ROC) curves and AUC values were generated and analyzed via the'pROC' package [21]. SHapley Additive exPlanation (SHAP) [22] values were used to assess feature importance in the best-performing model.

#### Mendelian randomization analysis

We performed two-sample Mendelian randomization (MR) analysis using large-scale European GWAS data to select instrumental variables (IVs). Details on GWAS selection are provided in the Supplementary Methods. For the labeling of harmonized glycan traits, we referenced the definitions provided by the TwinsUK study [23, 24]. IVs were chosen based on *p* value < 5 × 10^−^⁸ and limited to cis regions. Single-nucleotide polymorphisms (SNPs) in linkage disequilibrium (r^2^ > 0.01) and ambiguous palindromic SNPs (A/T or G/C alleles) were excluded. Outcome GWAS results were then extracted for the selected IVs. The Wald ratio test was used for single IV analyses, while the inverse variance weighted (IVW) method was applied for at least two IVs [25, 26]. Instrument heterogeneity and pleiotropy were assessed using Cochran’s Q test and MR-Egger regression. The core assumptions of MR—relevance, independence, and exclusion restriction—were considered and supported by the instrument selection strategy and statistical sensitivity tests. A significance threshold adjusted via the Benjamini‒Hochberg procedure was implemented to control the false discovery rate (FDR) to be < 0.05. Analyses were conducted by the R package “TwoSampleMR” (version 0.5.7) [27].

#### Glycan quantitative trait loci analysis

Glycan quantitative trait loci (Glycan-QTL) analysis was conducted in KORA FF4 using linear regression models implemented via the MatrixEQTL [28] R package, adjusting for age and sex. SNP filtering and quality control were followed by standard protocols using PLINK v2.0 [29]. Details of genotyping, imputation, and functional annotations are provided in the Supplementary Methods.

All analyses were conducted using Python (version 3.8.5), R statistics (version 4.3.3) and RStudio (version 2023.09.1 + 494).

## Results

### Characteristics of the KORA F4/FF4

The follow-up time for this study was 7 years. At baseline (F4), all participants were classified as NGT. Among the 473 participants in KORA FF4, 231 had prediabetes or were diagnosed with T2D, whereas 242 remained normoglycemic. In KORA F4, participants were categorized based on whether they were later diagnosed with prediabetes/T2D in FF4. Table 1 summarizes the baseline characteristics of the study participants in KORA F4, while Table 2 provides details specific to KORA FF4.

**Table 1:** Baseline characteristics of participants in KORA F4 (2006–2008)

	Total (*n* = 473)	Future NGT cases (*n* = 242)	Future prediabetes / T2D cases (*n* = 231)	*p* value^b^
	Mean (standard deviation) or number (%)			
Age (years)	57.4 ± 12.2	57.4 ± 12.3	57.3 ± 12.2	0.901
Sex, N (%) female	215 (45.5)	110 (45.5)	105 (45.5)	1
Body mass index (kg/m^2^)	27.9 ± 4.5	26.5 ± 4	29.3 ± 4.6	< 0.001
Waist circumference (cm)	95 ± 12.8	91 ± 11.9	99.2 ± 12.5	< 0.001
Waist height ratio	0.9 ± 0.1	0.9 ± 0.1	0.9 ± 0.1	< 0.001
Smoking status, N (%)				0.813
Current smoker	189 (40)	99 (40.9)	90 (39)	
Former smoker	211 (44.6)	108 (44.6)	103 (44.6)	
Never smoker	73 (15.4)	35 (14.5)	38 (16.5)	
Alcohol consumption (g/day)	15.7 ± 19.7	15.7 ± 19.2	15.7 ± 20.3	0.983
Physically active, N (%)	272 (57.5)	152 (62.8)	120 (51.9)	0.022
Systolic blood pressure (mm Hg)	123.3 ± 17.4	120.5 ± 16.5	126.2 ± 17.8	< 0.001
Diastolic blood pressure (mm Hg)	76.3 ± 9.6	74.6 ± 8.6	78.1 ± 10.3	< 0.001
Total cholesterol (mmol/L)	1.4 ± 0.3	1.5 ± 0.3	1.3 ± 0.3	< 0.001
Triglycerides (mmol/L) ^a^	3.6 ± 0.9	3.6 ± 0.8	3.7 ± 0.9	0.059
High‑density lipoprotein cholesterol (mmol/L)	1.2 (0.8, 1.7)	1.1 (0.7, 1.5)	1.4 (1, 1.9)	< 0.001
Low‑density lipoprotein cholesterol (mmol/L)	5.7 ± 1	5.7 ± 0.9	5.8 ± 1	0.276
HbA1c (%)	5.4 ± 0.3	5.3 ± 0.3	5.6 ± 0.3	< 0.001
HbA1c (mmol/mol)	36 ± 3	34 ± 4	38 ± 3	< 0.001
HOMA-IR	1.2 ± 0.7	0.9 ± 0.4	1.5 ± 0.9	< 0.001
HOMA-B (%)	90.9 ± 30.4	85.4 ± 22.3	96.6 ± 36.2	< 0.001
No-use of antidiabetic medications, N (%)	473 (100)	242 (100)	231 (100)	1
Cardiovascular diseases, N (%)	13 (2.7)	3 (1.2)	10 (4.3)	0.076
Urine albumin-to-creatinine ratio	15.1 ± 65	10.4 ± 24.5	19.9 ± 89.5	0.12
eGFRcr (ml/min/1.73 m^2^)	87.2 ± 15.3	88.2 ± 15.4	86.1 ± 15.1	0.136

Continuous variables are presented as the means ± SDs for normally distributed data and as medians (25th, 75th) for nonnormally distributed data. Categorical variables are presented as *n* (%)

^a^Reported as the median (interquartile range)

^b^*P* value was estimated by t test (continuous variables) or χ^2^ test (categorical variables)

^c^HbA1c, hemoglobin A1c; BMI, body mass index; WC, waist circumference; WHR, waist-height ratio; mm Hg, millimeters of mercury; HOMA-IR, homeostasis model assessment of insulin resistance; HOMA-B, homeostasis model assessment of β-cell function; T2D, type 2 diabetes; NGT, normal glucose tolerance; eGFRcr, estimated glomerular filtration rate based on serum creatinine

**Table 2:** Follow-up characteristics of participants in KORA FF4 (2013–2014)

	Total (*n* = 473)	NGT cases (*n* = 242)	T2D/prediabetes cases (*n* = 231)	*p* value ^b^
	Mean (standard deviation) or number (%)			
Age (years)	63.8 ± 12.3	63.8 ± 12.3	63.7 ± 12.3	0.93
Sex, N (%) female	215 (45.5)	110 (45.5)	105 (45.5)	1
Body mass index (kg/m^2^)	28.6 ± 5.1	26.9 ± 4.4	30.3 ± 5.2	< 0.001
Waist circumference (cm)	99.5 ± 14	94.7 ± 12.6	104.5 ± 13.6	< 0.001
Waist height ratio	0.9 ± 0.1	0.9 ± 0.1	0.9 ± 0.1	< 0.001
Smoking status, N (%)				0.793
Current smoker	202 (42.7)	101 (41.7)	101 (43.7)	
Former smoker	211 (44.6)	108 (44.6)	103 (44.6)	
Never smoker	60 (12.7)	33 (13.6)	27 (11.7)	
Alcohol consumption (g/day)	16.3 ± 20	16.6 ± 18.3	16.1 ± 21.6	0.81
Physically active, N (%)	264 (55.8)	156 (64.5)	108 (46.8)	< 0.001
Systolic blood pressure (mm Hg)	120.5 ± 17.3	118.7 ± 15.9	122.5 ± 18.5	0.018
Diastolic blood pressure (mm Hg)	73.5 ± 9.7	72.6 ± 8.4	74.5 ± 10.8	0.035
Total cholesterol (mmol/L)	1.7 ± 0.5	1.8 ± 0.5	1.5 ± 0.4	< 0.001
Triglycerides (mmol/L) ^a^	3.5 ± 0.9	3.5 ± 0.9	3.6 ± 1	0.6
High‑density lipoprotein cholesterol (mmol/L)	1.2 (0.9, 1.7)	1.1 (0.8, 1.4)	1.5 (1, 2)	< 0.001
Low‑density lipoprotein cholesterol (mmol/L)	5.6 ± 1	5.7 ± 1	5.6 ± 1.1	0.815
HbA1c (%)	5.6 ± 0.6	5.3 ± 0.3	5.8 ± 0.6	< 0.001
HbA1c (mmol/mol)	38 ± 6	34 ± 4	40 ± 6	< 0.001
HOMA-IR	1.4 ± 1	1 ± 0.5	1.8 ± 1.2	< 0.001
HOMA-B (%)	86.6 ± 32.9	84.7 ± 24.8	88.7 ± 39.6	0.194
Use of antidiabetic medications, N (%)	26 (5.5)	0 (0)	26 (11.3)	< 0.001
Cardiovascular diseases, N (%)	23 (4.9)	8 (3.3)	15 (6.5)	0.162
Urine albumin-to-creatinine ratio	16.9 ± 45.6	13 ± 31.6	21 ± 56.5	0.058
eGFRcr (ml/min/1.73 m^2^)	78.8 ± 16.1	79.6 ± 15.8	78 ± 16.4	0.288

Continuous variables are presented as the means ± SDs for normally distributed data and as medians (25th, 75th) for nonnormally distributed data. Categorical variables are presented as *n* (%)

^a^Reported as the median (interquartile range)

^b^*P* value was estimated by t test (continuous variables) or χ^2^ test (categorical variables)

^c^HbA1c, hemoglobin A1c; BMI, body mass index; WC, waist circumference; WHR, waist-height ratio; mm Hg, millimeters of mercury; HOMA-IR, homeostasis model assessment of insulin resistance; HOMA-B, homeostasis model assessment of β-cell function; T2D, type 2 diabetes; NGT, normal glucose tolerance; eGFRcr, estimated glomerular filtration rate based on serum creatinine

### Associations between glycans and prediabetes/T2D

Using the HILIC-UHPLC-FLD method, we analyzed the plasma N-glycome of 473 participants at two points from the KORA F4/FF4. In addition to analyzing differences in directly measured initial glycans, we examined derived traits, which represent averages of shared glycosylation features (including the degree of glycan branching, galactosylation, sialylation, and fucosylation) across various glycan structures (Supplementary Table 1) and are calculated from the initial glycan traits (Supplementary Table 2). To identify glycan changes associated with this progression, we employed a longitudinal model to compare differences between NGT and prediabetes/T2D patients.

**Fig. 1:**
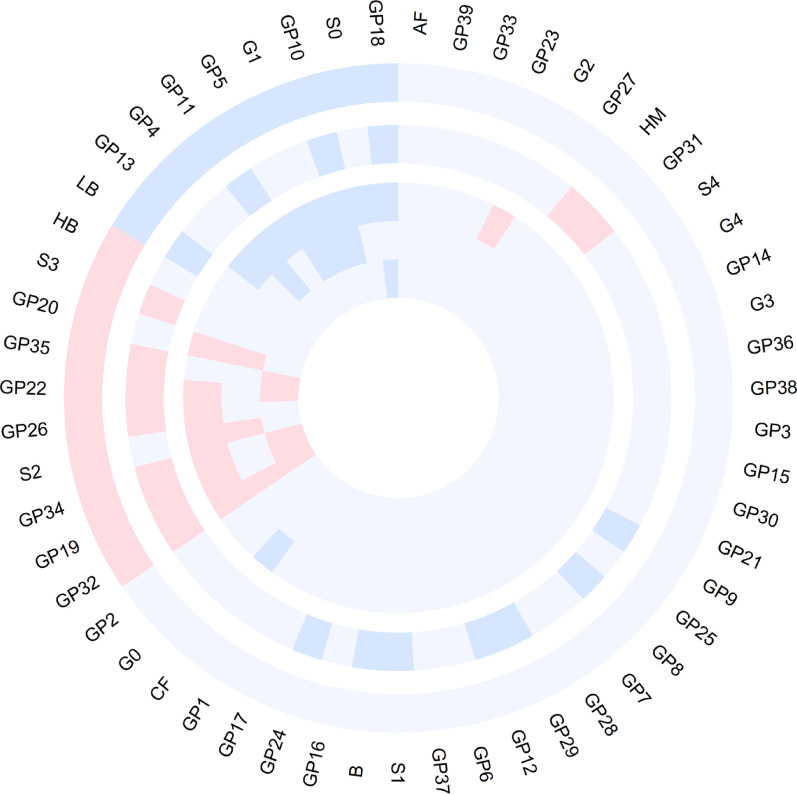
The circular plot displays glycan associations across 5 comparisons: from the outermost to the innermost circles, glycans differentiating NGT vs. prediabetes/T2D, glycans associated with BMI, glycans distinguishing NGT vs. T2D, glycans separating prediabetes vs. T2D, and glycans differentiating NGT vs. prediabetes. The red and blue segments represent positive and negative associations, respectively, with glycan names labeled around the outermost layer

Out of 39 directly measured glycans and 16 derived traits, 19 glycans demonstrated statistically significant differences in the basic model (adjusted for age, sex, and BMI). Among these, GP18, S0, GP5, G1, GP13, GP10, LB, GP4, and GP11 decreased, whereas GP32, GP19, GP34, S2, GP26, GP20, GP22, GP35, S3, and HB increased in prediabetes/T2D patients (Fig. 1; Supplementary Table 3). Further adjustment for smoking status, alcohol consumption, physical activity, HDL, and SBP in the full model revealed that 12 glycans (GP32, S2, GP35, GP19, GP34, GP22, GP20, GP26 increased, and S0, GP18, G1, GP5 decreased) remained significant (Supplementary Table 4). Notably, GP32 emerged as the most significant glycan, exhibiting a strong positive association with the progression of prediabetes/T2D.

### Sensitivity analyses

In the sensitivity analysis, we further examined glycan differences between subgroups: NGT versus prediabetes, NGT versus T2D, and prediabetes versus T2D (Fig. 1; Supplementary Tables 5 and 6). Among the 39 directly measured glycans and 16 derived traits, distinct patterns emerged across these comparisons, indicating progressive alterations in the plasma N-glycome. For example, in the basic model, glycans such as GP32, GP19, GP22, and GP34 exhibited a consistent upward trend from NGT to prediabetes and T2D, underscoring their potential as early indicators of disease progression. Conversely, GP18 demonstrated a downward trend, particularly during the transition from NGT to prediabetes and T2D. Additionally, in the transition from prediabetes to T2D, GP32 showed a notable upward trend. In the full model, the glycans GP32, GP22, GP34, and GP18 maintained these same trends, reinforcing their relevance in the progression of glucose dysregulation.

### Analysis of glycans related to diabetes traits

The LMM analysis revealed significant associations between the plasma N-glycome and BMI, as well as T2D-related traits, including FPG, HOMA-IR, and HbA1c. The inclusion of individual ID as a random effect accounted for intraindividual variability. In the basic model (adjusted for age and sex), 20 glycans demonstrated significant associations with BMI. Specifically, GP32, GP26, GP19, GP34, GP22, HM, GP35, GP31, and S3 were positively associated with BMI, whereas GP18, GP29, GP12, GP10, GP24, S1, LB, GP8, GP11, B, and GP9 were negatively associated with BMI (Fig. 1; Supplementary Table 7). Among these, 11 glycans (GP32, GP19, GP34, GP26, GP22, GP35, S3, LB, GP11, GP10, and GP18) were also associated with prediabetes/T2D in previous analyses.

Furthermore, GP26, GP32, and GP22 were significantly associated with all three T2D-related traits—FPG, HOMA-IR, and HbA1c (Supplementary Fig. 2; Supplementary Table 8). After adjusting for additional covariates, including smoking status, alcohol consumption, physical activity, HDL, and SBP, in the full model, GP32 and GP18 retained their significance for BMI, FPG, and HOMA-IR, demonstrating their robust and consistent associations with these traits (Supplementary Tables 9 and 10).

### Glycan-based diagnostic modeling and explainability at FF4

**Fig. 2:**
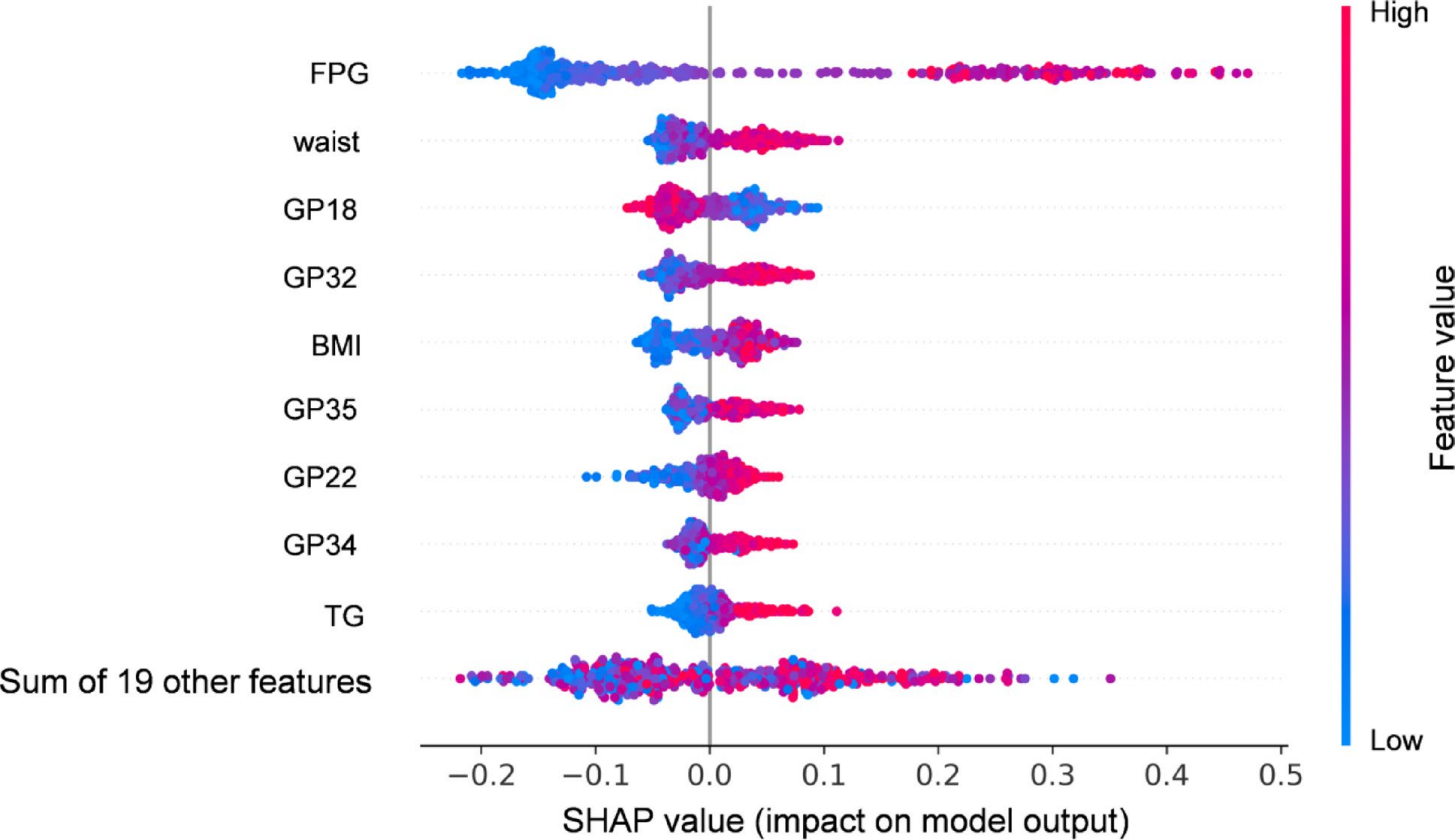
SHAP value plot illustrating the contributions of features to the RF classification model for prediabetes/T2D. The model incorporated 19 glycans and 9 FORS score components. Each point represents the SHAP value for a specific feature and its impact on the model output. The features are ranked by importance, with FPG, waist circumference, and GP18 and GP32 showing the highest contributions. Positive SHAP values indicate that higher feature values increase the probability of prediabetes/T2D, whereas negative SHAP values indicate that higher feature values act as protective factors, reducing the probability. The color gradient (blue to red) reflects feature values, with higher values in red and lower values in blue. This plot demonstrates the relative importance and direction of each feature's association with the model classification. *Abbreviations*: FPG, fasting plasma glucose; BMI, body mass index; TG, triglycerides; T2D, type 2 diabetes; SHAP, Shapley additive explanations; RF, random forest

Based on the KORA FF4 dataset, we developed RF models to classify glycemic status via three distinct sets of features: (1) 19 glycans associated with prediabetes/T2D; (2) 9 FORS variables, including age, sex, BMI, SBP, HDL, triglyceride (TG), parental history of diabetes, waist circumference, and FPG; (3) a combined model incorporating both 19 glycans and 9 FORS variables. Model optimization was tuned via tenfold cross-validation to determine the optimal hyperparameters. The classification performance, as assessed by the AUC, demonstrated that the glycan model yielded an AUC of 0.698 (0.603–0.793), the FORS model achieved an AUC of 0.822 (0.750–0.893), and the combined model outperformed the individual models, with an AUC of 0.895 (0.836–0.952) (Supplementary Table 11; Supplementary Fig. 3). To provide insights into the contributions of individual features within the combined model, SHAP values were calculated, which identified FPG, waist circumference, GP18, and GP32 as the most important contributors to the model’s classification accuracy (Fig. 2; Supplementary Table 12). These findings underscore the potential of integrating glycans with established clinical risk factors to enhance the diagnosis of IR/T2D.

### Mendelian randomization analysis

We conducted two-sample MR analysis for all glycans to explore the causal relationships between the plasma N-glycome and T2D, BMI, FPG, HOMA-IR, HbA1c (Supplementary Table 13), using glycan and derived traits GWAS summary statistics from the Twins UK dataset (n = 2763). Glycan ID mappings are provided in Supplementary Tables 14 and 15. GP19 (Wald ratio, β = − 0.208, FDR < 0.001), S1 (Wald ratio, β = 0.138, FDR = 0.002), GP18 (Wald ratio, β = − 0.045, FDR = 0.020) were the glycans for which we observed a statistically significant causal effect on T2D. GP19 (Wald ratio, β = − 0.063, FDR < 0.001), GP22 (Wald ratio, β = − 0.027, FDR < 0.001), GP36 (Wald ratio, β = − 0.030, FDR = 0.023) were significantly associated with BMI. For HbA1c, GP19 (Wald ratio, β = − 0.136, FDR < 0.001) and S1 (Wald ratio, β = − 0.055, FDR < 0.001) were significant. For the MR analysis of other traits, no evidence of a causal association was observed between the IVs and the respective outcomes after multiple testing was corrected. Additionally, we performed in the inverse direction; after harmonizing, no IVs remained for further analysis.

### Glycan quantitative trait loci analysis

In the KORA FF4 data, analysis was performed to identify associations between the plasma N-glycome and genetic variants, adjusting for age and sex. A total of 1,476,026 SNPs showed nominal significance (*p* < 0.05) across 19 glycans, and after FDR correction, 189 SNPs remained significant and were associated with four glycans (GP28, GP22, GP32, and GP34) (Supplementary Table 16). Notably, GP34 was associated with 142 SNPs. Among these, rs3967200, previously reported in the TwinsUK study [30], was confirmed to be associated with the plasma N-glycome. Further gene annotation revealed that GP34 is mostly associated with FUT8, RPL21P8, MIR625, and EIF1AXP2; GP18 is associated with ST3GAL4; GP22 is linked with FUT8; and GP32 is associated with ST3GAL4 (Supplementary Table 17). These findings highlight key loci and genes contributing to the genetic regulation of the plasma N-glycome.

## Discussion

In this study, we provide a comprehensive analysis of the plasma N-glycome and its associations with glucose metabolic dysregulation progression via longitudinal data from KORA F4/FF4. Importantly, plasma N-glycans were measured at both baseline (F4) and follow-up (FF4), allowing us to capture glycan alterations during the preclinical phase of diabetes development. Over a median follow-up of 7 years, we identified dynamic changes in specific glycans, particularly GP32 (triantennary trisialylated glycan A3G3S3), GP22 (biantennary, core fucosylated, disialylated FA2G2S2), GP34 (triantennary, core fucosylated, trisialylated FA3G3S3), and GP18 (biantennary, disialylated A2G2S2), which showed significant associations with the transition from normoglycemia to prediabetes and T2D. Glycans such as GP36 (tetraantennary, trisialylated A4G4S3), GP32, and GP22 were strongly associated with BMI and T2D-related traits, including FPG, HOMA-IR, and HbA1c, emphasizing their potential role in early diabetes pathophysiology. Classification models integrating glycans with established FORS factors in KORA FF4 demonstrated superior performance, with GP18 and GP32 showing high SHAP values, highlighting their critical contributions to model classification performance. Furthermore, MR analyses provided evidence of causal relationships between glycans and T2D, BMI, and HbA1c. Glycan–QTL analyses identified key genetic variants regulating plasma N-glycome traits, including associations between GP32 and ST3GAL4 and between GP34 and FUT8, shedding light on the genetic underpinnings of glycan–disease associations. These findings underscore the value of plasma N-glycans as diagnostic biomarkers for the early detection of glucose dysregulation and provide novel insights into their mechanistic roles in T2D pathophysiology.

Our analysis revealed that GP32 was the only glycan that was consistently significant across all the comparisons (Fig. 1). GP32 exhibited the most significant changes as NGT progressed to prediabetes and T2D and demonstrated high importance in the diagnostic classification model combining glycans with FORS factors. This finding is consistent with the TwinsUK study, which also identified GP32 as a significant marker for insulin resistance (IR) and T2D [14]. Additionally, GP32, along with GP19, was the only plasma glycan to significantly respond to a challenge meal in the PREDICT study, showing the strongest association with postprandial glucose spikes and fasting glucose levels [31]. GP32 is a glycan structure with trigalactosylation and trisialylation in a triantennary form (A3G3S3). The A3G3S3 structure in plasma mainly originates from α1-acid glycoprotein (AGP). AGP, also referred to as orosomucoid (ORM), is among the most abundant acute-phase proteins in humans and one of the most glycosylated proteins found in human plasma [32]. Previous studies have also confirmed that AGP levels are elevated in obese mice [33] and humans with metabolic syndrome and T2D [34, 35]. A recent study revealed that the AGP N-glycome profile could serve as a marker to distinguish individuals at risk for T2D [36].

The integration of glycan profiles with FORS factors significantly improved the diagnosis of prediabetes/T2D in KORA FF4, achieving an AUC of 0.895 in the combined model. This outperformed model is based solely on 19 glycans (AUC = 0.698) or FORS factors (AUC = 0.822). The superior performance of the combined model underscores the added value of incorporating glycomic data into existing diabetes risk assessment tools. Similar approaches have been explored in the prediction of future T2D, where multiomics integration enhances predictive accuracy [37]. Based on the prospective European Prospective Investigation of Cancer (EPIC)-Potsdam cohort (*n* = 27,548), a study demonstrated that the baseline plasma N-glycome (analyzed in 2,813 participants for T2D subset) effectively identified high-risk individuals ~ 6.5 years before T2D onset, achieving an AUC of 0.83 in distinguishing T2D patients from controls [15]. Our SHAP analysis further identified FPG, waist circumference, GP32, and GP18 as the most important classification-related features, highlighting the potential of these glycans as biomarkers for personalized diabetes risk assessment.

Using MR analysis, we identified causal links between specific glycans (GP19, S1, GP18) and T2D. Notably, high-mannose M9 glycan (GP19) had a negative causal effect on T2D risk, BMI, and HbA1c, suggesting a potential protective role. GP19 is thought to be derived primarily from apolipoprotein B-100 (ApoB-100) in plasma [38]. High-mannose glycans on vascular endothelial cells play critical roles in inflammation and leukocyte trafficking [39], which may explain their protective effect against T2D. The presence of GP19 in plasma may modulate inflammatory responses, thereby reducing the risk of metabolic dysfunction and diabetes progression. Although previous observational studies, including ours, reported positive associations between high-mannose glycans and T2D, our MR findings suggest a potential protective, context-dependent role of GP19. This discrepancy may reflect stage-specific effects, where GP19 could exert anti-inflammatory or compensatory effects during early metabolic dysregulation. Additionally, our glycan-QTL analysis revealed genetic loci (e.g., FUT8 and ST3GAL4) associated with plasma N-glycans, providing mechanistic insights into the genetic regulation of glycosylation. FUT8, a key enzyme in core fucosylation [40], regulates various biological processes, including immune response, signal transduction, proteasomal degradation, and energy metabolism [41]. Abnormal core fucosylation, which is mediated by FUT8, is frequently associated with the development of various cancers [42]. ST3GAL4, involved in α2,3-sialylation [43], has been shown to induce a selective increase in the expression of sialyl Lewis x (sLe^x^) [44]. The increase in sLe^x^ is associated with inflammatory infiltration and immune modulation within tumors [45], mechanisms that have also been demonstrated to be linked to diabetes [46, 47]. A study on proliferative diabetic retinopathy (PDR) demonstrated that high-glucose stimulation upregulated the expression of the sialyltransferase ST3GAL4 [48]. The identification of GP19 as a protective glycan and the role of FUT8 and ST3GAL4 in glycan metabolism have significant clinical implications. GP19 could serve as a potential biomarker for early diabetes detection and prevention. Additionally, targeting FUT8- and ST3GAL4-mediated glycosylation pathways may offer new therapeutic strategies for managing T2D and its complications. Although our MR and QTL analyses were performed independently, both converge to support the biological relevance of specific glycans and their genetic regulators in diabetes pathogenesis. This convergence of glycomic and genomic evidence aligns with the emerging perspective that glycans may serve as a “third layer” of biological information, complementing nucleic acids and proteins in cellular regulation and expanding our understanding of molecular mechanisms in complex diseases [49, 50].

Our study has several notable strengths. First, this study is the first to utilize two time-point glycomic measurements to investigate the dynamic changes in glycan levels during the 7-year transition from NGT to prediabetes/T2D. Second, in comparison with the TwinsUK study, we expanded the analysis by exploring the causal relationships between glycans and T2D, as well as related traits such as BMI, FPG, HOMA-IR, and HbA1c. Third, by integrating glycomic data with genetic data from the KORA S4, we provide novel insights into how genetic variations influence glycans associated with prediabetes/T2D. However, our study also has certain limitations. First, participants were selected based on data availability, without formal comparison to the rest of the F4 cohort, potentially introducing selection bias. Second, as our data were derived from individuals of European ancestry and lacked external longitudinal validation cohorts, the generalizability of our findings to other populations may be limited. Glycemic status was determined at two discrete time points approximately seven years apart, which may not fully capture transient glycemic changes between visits. Furthermore, the underlying mechanisms through which these glycans influence protein function and how glycans are causally altered at the genetic level remain poorly understood. To translate glycan profiling into clinical or public health applications, further efforts are needed in assay standardization, cost reduction, and reproducibility. However, this work is underway and significant effort is being invested in bringing glycomics closer to clinics.

## Conclusion

In conclusion, our longitudinal study highlights the critical role of the plasma N-glycome in the transition from normoglycemia to prediabetes and T2D. T2D, which is known to take years to develop and present symptoms, highlights the importance of early detection strategies. Our findings revealed that plasma N-glycome alterations preceded the onset of prediabetes and diabetes by several years, indicating their close connection to the pathophysiology of T2D. Incorporating glycans associated with prediabetes/T2D into the FORS model yielded valuable diagnostic classification performance. MR analysis further established a causal relationship between all plasma N-glycomes and T2D and associated metabolic traits, such as BMI and HbA1c. Additionally, glycan-QTL analysis sheds light on the genetic underpinnings of glycans linked to prediabetes/T2D, enhancing our understanding of the genetic influences on glycomic changes during disease progression. These insights offer new avenues for prevention, early diagnosis and targeted intervention.

## Electronic supplementary material

Below is the link to the electronic supplementary material.

Supplementary Material 1

Supplementary Material 2

## Data Availability

The KORA and the proteomics datasets are not publicly available but can be accessed upon application through the KORA-PASST use and access hub subject to KORA Board approval (https://helmholtz-muenchen.managed-otrs.com/external/).

## Abbreviations

2-AB: Dye 2-aminobenzamide
AGP: α1-Acid glycoprotein
ApoB-100: Apolipoprotein B-100
AUC: Area under the curve
BCAAs: Branched-chain amino acids
BMI: Body mass index
EPIC: European prospective investigation of cancer
FA: Fructosamine
FDR: False discovery rate
FORS: Framingham Offspring Risk Score
FPG: Fasting plasma glucose
GA: Glycated albumin
GEE: General estimating equation
glycan-QTL: Glycan quantitative trait loci
GPs: Glycan peaks
HbA1c: Hemoglobin A1c
HDL: High-density lipoprotein
HOMA-IR: Homoeostasis model assessment of insulin resistance
IFG: Impaired fasting glucose
IGT: Impaired glucose toler
IR: Insulin resistance
Ivs: Instrumental variables
KORA: Cooperative Health Research in the Region of Augsburg
L-GPC: Linoleoylglycerophosphocholine
LMM: Linear mixed model
LysoPC: Lysophosphatidylcholine
MR: Mendelian randomization
NGT: Normal glucose tolerance
OGTT: Oral glucose tolerance test
ORM: Orosomucoid
PDR: Proliferative diabetic retinopathy
RF: Random forest
ROC: Receiver operating characteristic
SBP: Systolic blood pressure
SDs: Standard deviations
SHAP: SHapley Additive exPlanation
sLe^x^: Sialyl Lewis x
SPE: Solid-phase extraction
T2D: Type 2 diabetes
UHPLC-FLR: Hydrophilic interaction ultrahigh-performance liquid chromatography with fluorescence detection
