# Supplementary material for: Longitudinal study reveals plasma glycans associations with prediabetes/type 2 diabetes in KORA study
Source: Cardiovasc Diabetol. 2025 Aug 6;24:321. doi: 10.1186/s12933-025-02853-y (PMC12329990; doi:10.1186/s12933-025-02853-y)
Supplement: Supplementary file 1 — Supplementary Material 1 [file 12933_2025_2853_MOESM1_ESM.docx]

**Supporting information**

**Longitudinal study reveals plasma glycans associations with prediabetes/type 2 diabetes in KORA study**

*Jiefei Niu ^1, 2, 3^*, Elke Rodriguez ^1, 2, 4^, Tamara Štambuk ^5^, Irena Trbojević-Akmačić ^5^, Nikol Mraz ^5^, Jochen Seissler ^4, 9^, Thomas Skurk ^7, 8^,* *Sabrina Schlesinger ^10, 11^, Annette Peters ^2, 4, 6^,* *Gordan Lauc ^5,12,^ *, Christian Gieger ^1, 2, 4,^ *, Harald Grallert ^1, 2, 4,^ **

*^1^* *Research Unit of Molecular Epidemiology, Helmholtz Zentrum München, 85764 Neuherberg, Germany*

*^2^ Institute of Epidemiology, Helmholtz Zentrum München, 85764 Neuherberg, Germany*

*^3^ Faculty of Medicine,* *Ludwig-Maximilians-University München, 81377 Munich, Germany*

*^4^ German Center for Diabetes Research (DZD), 85764 Neuherberg, Germany*

*^5^ Genos Ltd, Glycoscience Research Laboratory, Borongajska 83H, 10000 Zagreb, Croatia*

*^6^ Chair of Epidemiology, Faculty of Medicine, Ludwig-Maximilians-University München, 81377 Munich, Germany*

*^7^ School of Medicine, Technical University of Munich, 81675 Munich, Germany*

*^8^ ZIEL Institute for Food & Health, Core Facility Human Studies, Technical University of Munich, 85354 Freising, Germany*

*^9^ Medizinische Klinik und Poliklinik IV, Klinikum der Universität München, LMU, 80336 Munich, Germany*

*^10^ Institute for Biometrics and Epidemiology, German Diabetes Center, Leibniz Center for Diabetes Research at Heinrich Heine University Düsseldorf, Düsseldorf, Germany*

*^11^ German Center for Diabetes Research (DZD), Partner Düsseldorf, Muenchen-Neuherberg, Düsseldorf, Germany.*

*^12^ Faculty of Pharmacy and Biochemistry, University of Zagreb, Zagreb, Croatia*

**Corresponding authors: Jiefei Niu (jiefei.niu@helmholtz-munich.de), Gordan Lauc (glauc@genos.hr), Christian Gieger (christian.gieger@helmholtz-munich.de), Harald Grallert (harald.grallert@helmholtz-munich.de)*

**Supplementary Methods**

**Ascertainment of prediabetes and diabetes**

Subjects reporting a physician diagnosis of type 2 diabetes (T2D) or use of anti-diabetic medications during the follow-up were classified as incident diabetes cases only if their reports were validated by the treating physicians. For the remaining subjects, an oral glucose tolerance test (OGTT) with a 75g load of anhydrous glucose was conducted to ascertain diabetes status. OGTTs were performed in the morning hours (7:00- 11:00 h). Subjects were instructed to fast for 10 hours overnight, to avoid heavy physical activity and not to smoke before or during the OGTT. The incident NGT, prediabetes, or T2D were defined based on OGTT or a validated physician diagnosis.

The classification of prediabetes and T2D followed the 1999/2006 WHO criteria (1). Normoglycaemia was characterized by fasting glucose levels below 6.1 mmol/L and 2-hour glucose levels under 7.8 mmol/L. Prediabetes encompassed three categories: isolated impaired fasting glucose (IFG), with fasting glucose between 6.1 mmol/L and 7.0 mmol/L and 2-hour glucose below 7.8 mmol/L; isolated impaired glucose tolerance (IGT), with fasting glucose below 6.1 mmol/L and 2-hour glucose ranging from 7.8 mmol/L to less than 11.1 mmol/L; and combined IFG and IGT. Newly diagnosed diabetes was identified by fasting glucose of 7.0 mmol/L or higher or 2-hour glucose of 11.1 mmol/L or above.

**Clinical measurements and assessments of risk factors**

In the Cooperative Health Research in the Region of Augsburg (KORA) F4 study, glucose levels were measured using the hexokinase method (GLU Flex, Dade Behring, Deerfield, Illinois, USA). In the KORA FF4 study, glucose was assessed using an enzymatic colorimetric method (GLU assay) on a Dimension Vista 1500 instrument (Siemens Healthcare Diagnostics Inc., Newark, USA) or the GLUC3 assay on a Cobas c702 instrument (Roche Diagnostics GmbH, Mannheim, Germany). Insulin levels in KORA F4 were determined via an electrochemiluminescence immunoassay on a Cobas e602 instrument (Roche Diagnostics GmbH, Mannheim, Germany). In KORA FF4, insulin was evaluated either through a solid-phase enzyme-labeled chemiluminescent immunometric assay on an Immulite 2000 system (Siemens Healthcare Diagnostics Inc., Newark, USA) or using an electrochemiluminescence immunoassay on a Cobas e602 instrument (Roche Diagnostics GmbH, Mannheim, Germany) (2, 3). Details regarding the calibration of FF4 measurements performed with the methods of Siemens and Roche can be found elsewhere (3). Hemoglobin A1C (HbA1c) was measured by cation-exchange high-performance liquid chromatography using the Adams HA 8160 Hemoglobin Analysis System (Arkray, distributed by A. Menarini Diagnostics, Florence, Italy) in KORA F4 and the Variant II Turbo HbA1c Kit-2.0 (BioRad Laboratories, Hercules, USA) in KORA FF4. HDL cholesterol, LDL cholesterol, total cholesterol (TCHO), triglycerides (TG), and fasting plasma glucose (FPG) were assessed. A detailed overview of used laboratory methods is provided in the previous (4).

Additionally, the homeostasis model assessment of insulin resistance (HOMA-IR) was determined using the formula: (fasting insulin (mU/l) ×fasting glucose (mmol/l))/22.5. Beta-cell function, as assessed by the homeostasis model assessment of beta-cell function (HOMA-B), was calculated using the formula: (fasting insulin (mU/l) ×20)/ (fasting glucose (mmol/l)–3.5).

In all KORA studies, trained staff conducted standardized face-to-face interviews, during which they collected the following information: age, sex, weight, height, waist circumference (WC), waist-hip ratio (WHR), physical activity (active/ inactive), smoking status (never/former/current smoker), alcohol consumption, and fasting status (fasting for 8 h or more before blood was taken, yes/no). WC was evaluated at the minimum abdominal girth. Body weight and height were measured in light clothing by trained investigators. Body mass index (BMI) was calculated as body weight (kg) divided by the square of height (m). Anthropometric indices and blood pressure (BP) were measured based on standard protocols. Blood pressure (BP) was measured three times on the right arm in a sitting position after a fifteen-minute rest using an automatic device (HEM-705CP, Fa. OMRON HEALTHCARE GmbH) and the mean of the second and third measurements were used for analysis. Triglycerides were measured using the Boehringer GPO-PAP assay (4). Physical activity was assessed through a questionnaire that asked about weekly exercise frequency and duration during both summer and winter, with responses categorized into two groups: active and inactive (5). A participant was considered active if they engaged in sports during both summer and winter for more than one hour per week in at least one of the seasons. Smoking status was classified into three categories: never smoked, former smoker, and current smoker. The parental history of diabetes was also assessed and was defined as either maternal or paternal diabetes or both. Participants were also asked to bring the original packaging of any pharmaceutical products they had taken in the last 7 days before the examination. Additionally, the use of medications was classified according to the Anatomical Therapeutic Chemical Classification System (ATC) codes (4).

**Plasma N-glycome measurements**

**Release of Total Plasma Protein N-Glycans：**Plasma samples (10 μL) were denatured by adding 20 μL of 2% (w/v) sodium dodecyl sulfate (SDS, Invitrogen, USA) and incubating at 65°C for 10 minutes. Subsequently, 10 μL of 4% (v/v) Igepal CA-630 (Sigma-Aldrich, USA) was added, and the mixture was shaken for 15 minutes using a plate shaker (Gujarat Fluorochemicals Limited, Germany). N-glycans were released by incubating the samples overnight at 37°C with 1.2 U PNGase F (Promega, USA).

**Labeling and HILIC-Solid Phase Extraction of Released N-Glycans：**The released N-glycans were labeled with 2-aminobenzamide (2-AB), a fluorescent dye. The labeling solution was prepared in-house by dissolving 2-AB (19.2 mg/mL) and 2-picoline borane (44.8 mg/mL) in a mixture of dimethyl sulfoxide (Sigma-Aldrich) and glacial acetic acid (Merck, Germany) at a 70:30 (v/v) ratio. Each sample was incubated with 25 μL of this labeling solution at 65°C for 2 hours. To achieve a final acetonitrile concentration of 96%, 700 μL of cold 100% acetonitrile was added. Samples were then transferred to a 0.2 μm hydrophilic polypropylene membrane (GHP) filter plate (Pall Corporation, USA). Before sample loading, the filter plate was prewashed with 70% ethanol and water, then equilibrated with 96% acetonitrile. Solvent and impurities, including excess labeling dye and reducing agent, were removed using HILIC solid-phase extraction (SPE) on the filter plate with a vacuum manifold (Millipore Corporation, USA). Samples were washed five times with 96% acetonitrile, and the labeled N-glycans were eluted twice with 90 μL of ultrapure water and stored at −20°C until analysis.

**HILIC-UPLC-FLR Profiling of Total Plasma Protein N-Glycans：**Fluorescently labeled N-glycans were separated using hydrophilic interaction liquid chromatography (HILIC) on an Acquity ultra-performance liquid chromatography (UPLC) H-Class system (Waters, Milford, USA) equipped with a quaternary solvent manager, sample manager, and fluorescence detector set to excitation and emission wavelengths of 250 nm and 428 nm, respectively. The system was controlled via Empower 3 software (Waters). Chromatographic separation was performed using a Waters BEH Glycan column, with 100 mM ammonium formate (pH 4.4) as solvent A and liquid chromatography-mass spectrometry (LC-MS) grade acetonitrile as solvent B. Each 96-well plate included five standard samples and one blank for quality control and batch correction. The separation was achieved using a linear gradient of 70%–53% acetonitrile at a flow rate of 0.561 mL/min over 25 minutes. Calibration was conducted using hydrolyzed and 2-AB-labeled glucose oligomers, allowing retention times of glycans to be expressed in glucose units. Chromatograms were automatically processed to generate 39 glycan peaks (GP1–GP39).

A detailed description of the glycan structures regarding each glycan peak is provided in Supplementary Table 1. Glycan peaks were identified according to their elution positions, measured in glucose units, and compared to reference data from the GlycoStore database (https://glycostore.org/) for structural assignment. In addition to the 39 directly measured plasma glycan traits, 16 derived traits were calculated. These derived traits represent the average levels of specific glycosylation features, such as branching, sialylation, galactosylation, fucosylation, and the presence of bisecting N-acetylglucosamine (GlcNAc). The formulas used for calculating the derived traits are detailed in Supplementary Table 2.

**Genotyping and Glycan-QTL Analysis**

Genotyping was conducted using the Affymetrix Axiom Chip (6, 7). Information on the position of SNPs and their genetic features was obtained from the UCSC Genome Browser on the human genome [February 2009 (GRCh37/hg19)] (8). Prephasing was performed using SHAPEIT v2, and imputation was carried out with IMPUTE v2.3.0, using the 1000 Genomes Project (phase 1 integrated haplotypes CEU) as the reference panel. Single-nucleotide polymorphisms (SNPs) were filtered to exclude non-monomorphic variants, those with a call rate lower than 98%, and SNPs that deviated significantly from Hardy-Weinberg Equilibrium (p < 5 × 10^−6^) were also excluded. Additionally, SNPs with imputation quality scores below 30%, as defined by IMPUTE, were further excluded. Further filtering criteria applied in this study included the exclusion of SNPs with a minor allele frequency (MAF) of less than 1%. Filtering was carried out using PLINK version 2.0 (9). Following these criteria, a total of 7,799,795 SNPs remained. All individuals included in the analysis were European ancestry. Genome-wide association analysis (GWAS) was performed using KORA FF4 (n = 442) for 13 glycans and 6 derived traits associated with prediabetes/T2D. The MatrixEQTL (10) 2.2 R package was used to calculate glycan quantitative trait loci (QTL). Values for 19 glycans in FF4 were transformed to a standard normal distribution by R package GenABEL (11) before model fitting. Age and sex were included as covariates in the model. Multiple testing correction was performed using the Benjamini-Hochberg (BH) method, with genome-wide statistical significance defined as a false discovery rate (FDR) < 0.05.Functional annotations for significant SNPs were performed using the Ensembl Variant Effect Predictor (VEP) online tool (https://grch37.ensembl.org/Homo_sapiens/Tools/VEP) (12), enabling the identification of SNPs' potential biological impact.

**Detailed description of two-sample Mendelian randomization (MR)**

Specifically, we extracted the instrumental variables (IVs) for the glycans from the GWAS database: Twins UK dataset (n = 2,763) (13, 14). For T2D GWAS, we obtained IVs from the dataset “ebi-a-GCST90018926” (15) (38,841 cases and 451,248 controls from European ancestry). For continuous outcomes, we used GWAS data from “ukb-b-19953” for BMI (n = 461,460), “ieu-b-118” (16) (n = 37,037) for HOMA-IR, “ebi-a-GCST90002232” (17) (n = 200,622) for fasting glucose, and “ebi-a-GCST90014006” (18) (n = 389,889) for HbA1c. All datasets were derived from European ancestry populations, with priority given to databases with the largest sample sizes.

**Supplementary Tables** (see separate Excel file)


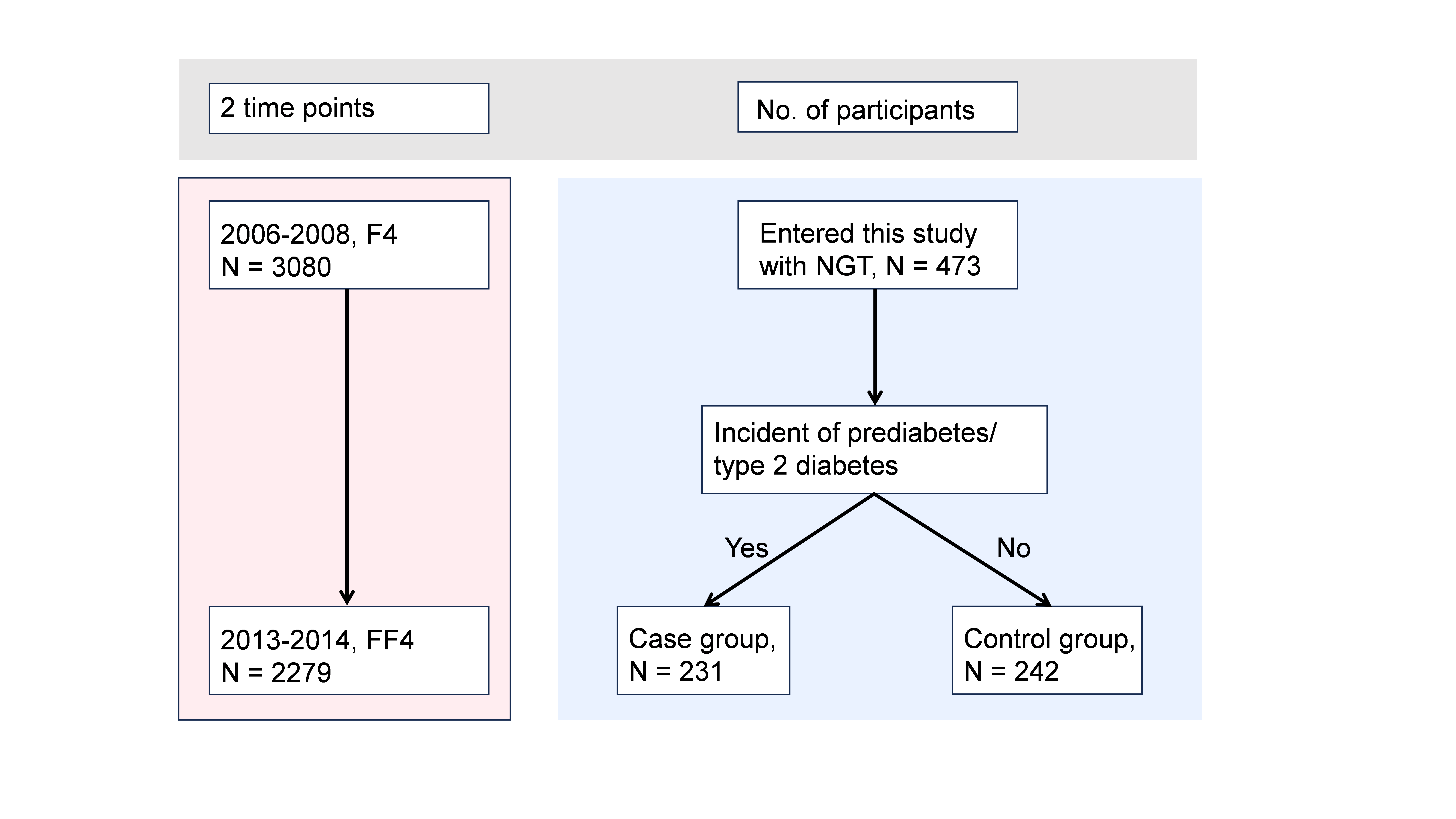
**Supplementary Figures**

**Supplementary Figure 1.** Flowchart of study design. The study included 2 time points: F4 (2006–2008, N = 3,080) and FF4 (2013–2014, N = 2,279). Among 473 participants with normal glucose tolerance (NGT) at baseline, 231 developed T2D or prediabetes during follow-up (case group), while 242 did not (control group).

Abbreviation: NGT, normal glucose tolerance.


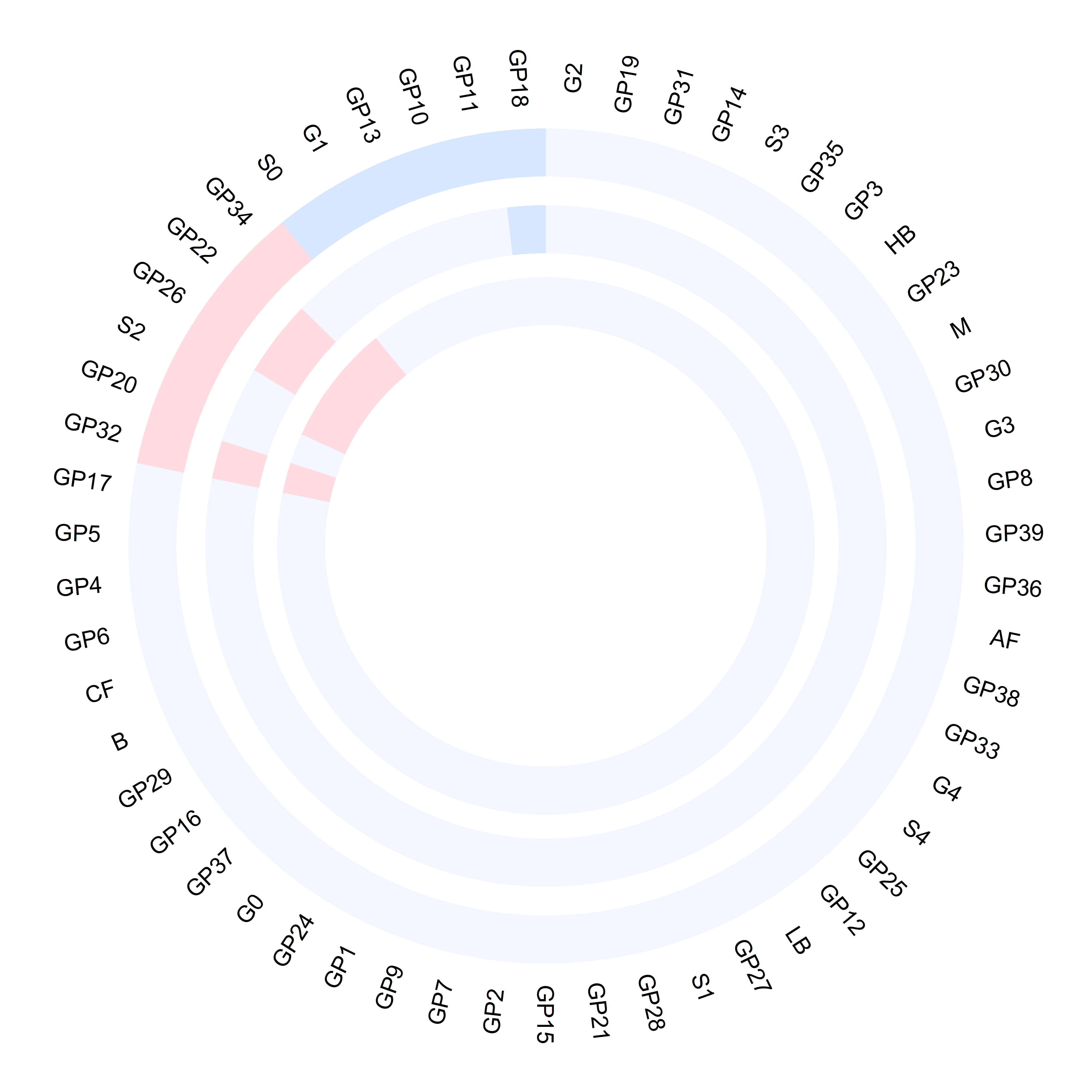


**Supplementary Figure 2.** The circular plot displays glycan associations T2D related traits (FPG, HOMA-IR, HbA1c): from the outermost to the innermost circles, glycans related with FPG, glycans associated with HOMA-IR, glycans distinguishing with HbA1c. Red and blue segments represent positive and negative associations, respectively, with glycans names labeled around the outermost layer.

**
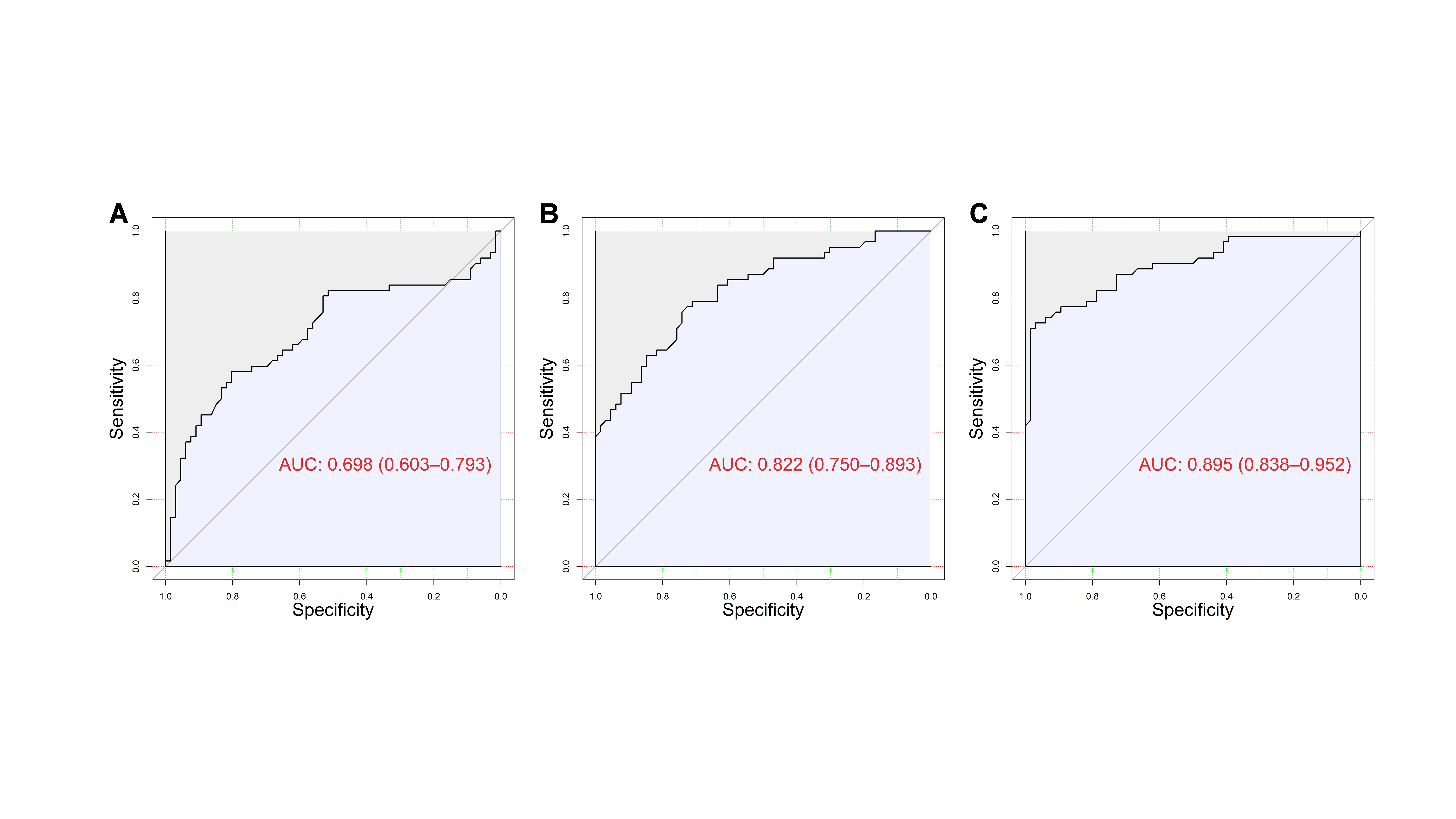
Supplementary Figure 3.** The figure shows the classification performance of 3 models for prediabetes/T2D using KORA FF4 dataset: (1) a glycans-only model based on 19 glycans associated with prediabetes/T2D, (2) a clinical risk factors (FORS) model, and (3) a combined model integrating both glycans and clinical risk factors (FORS). The classification performance, assessed by the area under the curve (AUC), demonstrates that the glycans-only model achieved an AUC of 0.698 (0.603-0.793), the FORS model yielded an AUC of 0.822 (0.750-0.893), and the combined model outperformed both with an AUC of 0.895 (0.836-0.952).

**References**

1. Alberti KG, Zimmet PZ. Definition, diagnosis and classification of diabetes mellitus and its complications. Part 1: diagnosis and classification of diabetes mellitus provisional report of a WHO consultation. Diabet Med. 1998;15(7):539-53.

2. Rathmann W, Haastert B, Icks A, Löwel H, Meisinger C, Holle R, et al. High prevalence of undiagnosed diabetes mellitus in Southern Germany: target populations for efficient screening. The KORA survey 2000. Diabetologia. 2003;46(2):182-9.

3. Huth C, von Toerne C, Schederecker F, de Las Heras Gala T, Herder C, Kronenberg F, et al. Protein markers and risk of type 2 diabetes and prediabetes: a targeted proteomics approach in the KORA F4/FF4 study. Eur J Epidemiol. 2019;34(4):409-22.

4. Laxy M, Knoll G, Schunk M, Meisinger C, Huth C, Holle R. Quality of diabetes care in germany improved from 2000 to 2007 to 2014, but Improvements diminished since 2007. Evidence from the population-based KORA studies. Plos One. 2016;11(10):e0164704.

5. Meisinger C, Thorand B, Schneider A, Stieber J, Döring A, Löwel H. Sex differences in risk factors for incident type 2 diabetes mellitus: the MONICA Augsburg cohort study. Arch Intern Med. 2002;162(1):82-9.

6. Livshits G, Macgregor AJ, Gieger C, Malkin I, Moayyeri A, Grallert H, et al. An omics investigation into chronic widespread musculoskeletal pain reveals epiandrosterone sulfate as a potential biomarker. Pain. 2015;156(10):1845-51.

7. Ha NT, Freytag S, Bickeboeller H. Coverage and efficiency in current SNP chips. Eur J Epidemiol. 2014;22(9):1124-30.

8. Kent WJ, Sugnet CW, Furey TS, Roskin KM, Pringle TH, Zahler AM, et al. The human genome browser at UCSC. Genome Res. 2002;12(6):996-1006.

9. Chang CC, Chow CC, Tellier LC, Vattikuti S, Purcell SM, Lee JJ. Second-generation PLINK: rising to the challenge of larger and richer datasets. Gigascience. 2015;4:7.

10. Shabalin AA. Matrix eQTL: ultra fast eQTL analysis via large matrix operations. Bioinformatics. 2012;28(10):1353-8.

11. Aulchenko YS, Ripke S, Isaacs A, van Duijn CM. GenABEL: an R library for genome-wide association analysis. Bioinformatics. 2007;23(10):1294-6.

12. McLaren W, Gil L, Hunt SE, Riat HS, Ritchie GR, Thormann A, et al. The ensembl variant effect predictor. Genome Biol. 2016;17(1):122.

13. Sharapov SZ, Tsepilov YA, Klaric L, Mangino M, Thareja G, Shadrina AS, et al. Defining the genetic control of human blood plasma N-glycome using genome-wide association study. Hum Mol Genet. 2019;28(12):2062-77.

14. Sharapov S, Tsepilov Y, Klaric L, Mangino M, Thareja G, Simurina M, et al. Genome-wide association summary statistics for human blood plasma glycome. 1 ed: Zenodo.

15. Sakaue S, Kanai M, Tanigawa Y, Karjalainen J, Kurki M, Koshiba S, et al. A cross-population atlas of genetic associations for 220 human phenotypes. Nat Genet. 2021;53(10):1415-24.

16. Dupuis J, Langenberg C, Prokopenko I, Saxena R, Soranzo N, Jackson AU, et al. New genetic loci implicated in fasting glucose homeostasis and their impact on type 2 diabetes risk. Nat Genet. 2010;42(2):105-16.

17. Chen J, Spracklen CN, Marenne G, Varshney A, Corbin LJ, Luan J, et al. The trans-ancestral genomic architecture of glycemic traits. Nat Genet. 2021;53(6):840-60.

18. Mbatchou J, Barnard L, Backman J, Marcketta A, Kosmicki JA, Ziyatdinov A, et al. Computationally efficient whole-genome regression for quantitative and binary traits. Nat Genet. 2021;53(7):1097-103.
